# Synthetic Aperture Imaging Using High-Frequency Convex Array for Ophthalmic Ultrasound Applications

**DOI:** 10.3390/s21072275

**Published:** 2021-03-24

**Authors:** Hae Gyun Lim, Hyung Ham Kim, Changhan Yoon

**Affiliations:** 1 Department of Biomedical Engineering, Pukyong National University, Busan 48513, Korea; hglim@pknu.ac.kr; 2 Department of Convergence IT Engineering, Pohang University of Science and Technology, Pohang 37673, Korea; 3 Department of Biomedical Engineering, Inje University, Gimhae 50834, Korea; 4 Department of Nanoscience and Engineering, Inje University, Gimhae 50834, Korea

**Keywords:** high-frequency ultrasound, ophthalmic imaging, synthetic aperture, convex array transducer

## Abstract

High-frequency ultrasound (HFUS) imaging has emerged as an essential tool for pre-clinical studies and clinical applications such as ophthalmic and dermatologic imaging. HFUS imaging systems based on array transducers capable of dynamic receive focusing have considerably improved the image quality in terms of spatial resolution and signal-to-noise ratio (SNR) compared to those by the single-element transducer-based one. However, the array system still suffers from low spatial resolution and SNR in out-of-focus regions, resulting in a blurred image and a limited penetration depth. In this paper, we present synthetic aperture imaging with a virtual source (SA-VS) for an ophthalmic application using a high-frequency convex array transducer. The performances of the SA-VS were evaluated with phantom and ex vivo experiments in comparison with the conventional dynamic receive focusing method. Pre-beamformed radio-frequency (RF) data from phantoms and excised bovine eye were acquired using a custom-built 64-channel imaging system. In the phantom experiments, the SA-VS method showed improved lateral resolution (>10%) and sidelobe level (>4.4 dB) compared to those by the conventional method. The SNR was also improved, resulting in an increased penetration depth: 16 mm and 23 mm for the conventional and SA-VS methods, respectively. Ex vivo images with the SA-VS showed improved image quality at the entire depth and visualized structures that were obscured by noise in conventional imaging.

## 1. Introduction

High-frequency ultrasound (HFUS) imaging (>15 MHz) has evolved rapidly over the last decade and opened up new applications such as ophthalmic, dermatologic, intravascular, small animal, and molecular imaging [1,2,3,4,5,6,7]. It can provide sub-millimeter spatial resolution determined by $f_{\#}\cdot \lambda$ (where $f_{\#}$ is defined as a ratio of a focal distance to a length of the aperture used for transmission/reception, and λ is the wavelength) at the expense of a shallow penetration depth. Most custom-built or commercialized HFUS imaging systems have employed mechanically scanning single-element transducers to form an image [8,9,10]. While these single-element imaging systems have offered exciting potential for many applications, they suffered from low spatial resolution and signal-to-noise ratio (SNR) in the out-of-focus regions, thus deteriorating the image quality [11]. In addition, mechanical scanning limits the frame rate.

The adoption of array transducers in HFUS imaging has allowed for improving the spatial resolution and SNR [9,12,13]. The array transducer-based systems capable of dynamic receive focusing use electronic scanning to form an image; thus it provides a higher frame rate and image quality than those by the mechanical scanning systems. Although it can enhance the overall image quality of HFUS, two-way focusing is only achieved at the vicinity of the transmit focal depth. To mitigate this, multi-zone transmit focusing, where transmit focusing is conducted at two or more depths for each scanline at the expense of frame rate (reduced by a factor of the number of transmitting foci), has been used [14]. In addition to the problem of one-way dynamic focusing, the HFUS imaging still suffers from low SNR due to diffraction and frequency-dependent attenuation that linearly increases with frequency [15]. Coded excitation can be a solution for improving the SNR [16,17]. However, the spatial resolution is still limited by the diffraction of the wave.

A viable solution to obtain a high spatial resolution, SNR, and frame rate is to employ synthetic aperture (SA) imaging techniques that are based on the superposition of unfocused transmit wave fields. Several different SA methods have been proposed and have shown their ability to enhance image quality at the expense of computational cost [18,19,20,21]. Among them, multi-element SA with a virtual source (SA-VS) that can achieve high SNR with full two-way dynamic focusing would be the most prominent method [20]. Clinical evaluations of the SA-VS on cancer diagnosis over conventional imaging were performed [22,23]. It was demonstrated that the improved image quality could be obtained using the SA-VS method and was perceived by radiologists. Recently, efficient architectures for SA-VS imaging have been proposed and implemented in prototype systems [21,24]. In addition, recent advances in graphic processor unit (GPU) computing in medical ultrasound imaging may facilitate more rapid commercialization of SA techniques [25,26].

The purpose of this study was to evaluate the feasibility of the SA-VS for ophthalmic imaging using a high-frequency convex array transducer by demonstrating its effectiveness in enhancing image quality compared to the conventional one-way dynamic focusing. Note that the high-frequency convex array transducer is the only transducer, and this is the first time we applied synthetic aperture imaging using this transducer for ophthalmic imaging. The main advantage of a convex array is that it can image the whole posterior segment at once. The performances of SA-VS were evaluated through phantoms and ex vivo experiments. Pre-beamformed radio-frequency data were acquired by using a custom-built research system. In the phantom experiments, spatial resolution and SNR were quantitatively assessed and compared with the conventional dynamic receive focusing method. In addition, an excised bovine eye was scanned, and the SA-VS image showed improved image quality.

## 2. Methods

### 2.1. Principle of Synthetic Aperture Imaging with a Virtual Source

**Figure 1:**
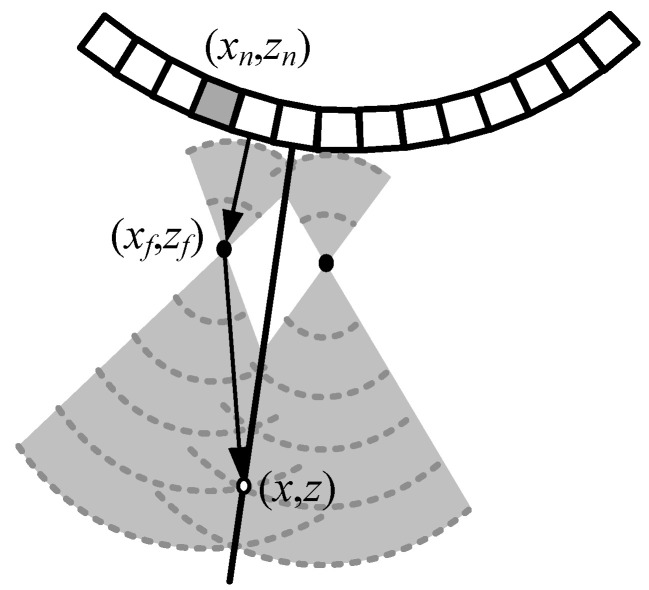
Principle of the transmit field synthesis in the synthetic aperture with a virtual source (SA-VS) imaging which represents the same sequences of transmit/receive as in the conventional imaging method.

Figure 1 shows the principle of synthesizing transmit fields in SA-VS imaging, which is capable of achieving two-way dynamic focusing at all imaging depths. A detailed description of SA-VS can be founded in [20]. Here, we briefly introduce the SA-VS. The SA-VS uses the same transmission (focused transmit) and reception procedures as in the conventional B-mode imaging. Thus, the frame rate of SA-VS is identical to that of the conventional method. In the SA-VS imaging method, a virtual source is regarded to be located at a transmit focal point where spherical waves assume to propagate from it. As can be seen, two transmit fields from different sub-apertures pass an imaging point, (x,z). Thus, the transmit focusing delay, $\tau_{tx}$, for an imaging point can be obtained by computing the arrival time of wavefront, given by

(1) $$\tau_{tx}\left(x,z\right)=\frac{z_{tx}\pm \sqrt{{\left(x_{f}-x\right)}^{2}+{\left(z_{f}-z\right)}^{2}}}{c},$$

where $z_{tx}$ is the transmit focal depth, $\left(x_{f},z_{f}\right)$ is the Cartesian coordinates of the transmit focal point, and *c* is the propagating speed of sound in soft tissue. The positive and negative signs in (1) are, respectively, applied in the areas after and before the transmit focal point. The receive focusing delay of *n*th element, $\left(x_{n},z_{n}\right)$, for the imaging point is identical to that in the conventional dynamic focusing method, which is computed by

(2) $$\tau_{n,RX}\left(x,z\right)=\frac{\sqrt{{\left(x-x_{n}\right)}^{2}+{\left(z-z_{n}\right)}^{2}}}{c}.$$

Based on these delays, the beamforming of the SA-VS can be achieved by

(3) $$A\left(x,z\right)=\overset{M}{\underset{m=-M}{\sum }}\overset{N-1}{\underset{n=0}{\sum }}a_{n}\cdot r_{m,n}\left(t-\left(\tau_{TX}\left(x,z\right)+\tau_{n,RX}\left(x,z\right)\right)\right),$$

where $a_{n}$ is the apodization function, $r_{m,n}\left(t\right)$ is the radio-frequency (RF) data received by the *n*th element for the *m*th scanline, 2*M* + 1 is the number of sub-apertures used in synthesizing, and *N* is the number of channels at each sub-aperture.

As can be seen in Figure 1, the number of scanlines that can be used for synthesizing varies according to the imaging depth. At the transmit focal depth, there is no scanline that can be synthesized. However, the number of scanlines incorporated in the transmit field synthesis increases as the imaging point moves away from the transmit focal depth, resulting in an increment of signal strength after synthesis. Thus, it requires a compensation method in the SA-VS method to obtain uniform brightness similar to those in the conventional method. For this, the beamformed RF signal in the SA-VS is divided by $\sqrt{M_{s}}$ where $M_{s}$ is the number of scanlines actually used for synthesis at a certain depth. This can be done by incorporating the values, $1/\sqrt{M_{s}}$, in the apodization function in (3).

### 2.2. Experimental Setup and Evaluation Metrics

To evaluate the performances, pre-beamformed RF data from phantoms and ex vivo bovine eye were acquired using a 64-channel research imaging system developed in our laboratory [13]. The system is composed of 256-channel of analog front-end pulser/receiver, 64-channel of time-gain compensation (TGC), and an analog-to-digital converter (ADC) with 12-bit resolution. A custom-built 20 MHz high-frequency transducer made with 1–3 composites was used in the experiments [27]. The array consists of 192 elements with an element pitch of 111 µm, and a −6 dB fractional bandwidth was 64%. The pre-beamformed RF data sampled at 100 MHz were stored in field-programmable gate arrays (FPGAs) that are embedded in the system and transferred to a PC. Off-line processing using MATLAB (MathWorks Inc., Natick, MA, USA) was carried out. The lateral resolutions were measured with 20 µm tungsten wire targets located at each depth. An agar phantom was made to estimate the SNR of both imaging methods, i.e., conventional and SA-VS. For ex vivo experiments, an excised bovine eye was purchased from Sierra Medical Inc. (Whittier, CA, USA). The eye was immersed in deionized water and fixed on a custom-made holder while scanning. In both beamformations (conventional and SA-VS), the optimal sound speed was estimated to minimize the effect from phase aberration artifacts, which is a first-order solution for phase aberration correction [28].

For quantitative comparison, −6 dB lateral resolutions were measured from the conventional and SA-VS images. In addition, the SNR as a function of depth was calculated by

(4) $$SNR\left(z\right)=10log10\left(\frac{P_{echo}\left(z\right)}{P_{noise}\left(z\right)}\right),$$

where *P_echo_* and *P_noise_* are the mean power of echo and noise signals along with the imaging depth (*z*), respectively. The mean power at each depth was computed by summing the envelope signal laterally at the speckle region. The system noise was measured by acquiring pre-beamformed RF data without transmission. The acquired noise signal was processed in the same manner for each method, i.e., conventional and SA-VS methods. Based on the SNR, the penetration depths defined as the depth where SNR falls below 0 dB were estimated.

## 3. Results and Discussion

**Figure 2:**
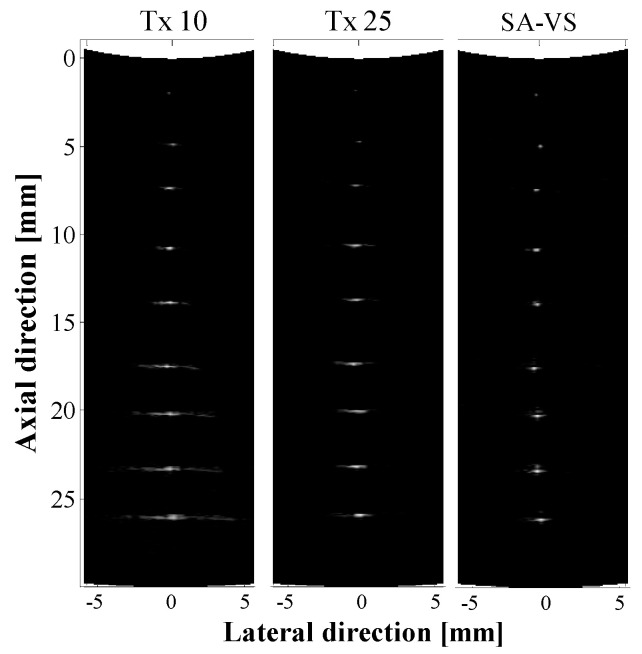
B-mode images of wire targets located at each depth obtained by the conventional method with transmitting focal depths of 10 mm (Tx 10), 25 mm (Tx 25), and the SA-VS method, respectively. The number of sub-aperture used in synthesis in the SA-VS was 33.

Figure 2 shows B-mode images of wire targets generated by the conventional and SA-VS methods, respectively. Two images were acquired for the conventional imaging with different transmit focal depths, 10 (Tx10) and 25 mm (Tx25), which are shown in Figure 2a,b, respectively. In the SA-VS imaging, the transmit focal depth was 10 mm to maximize the number of synthesizable sub-aperture at far depth. Note that the number of sub-aperture for synthetic aperture varies with different focal depth (see Figure 1). Since the main imaging target of the high-frequency convex array transducer is the posterior segment of the eye, the transmit focal depth of 10 mm was chosen. The maximum number of sub-aperture used in synthesis, in (3), was calculated based on the configuration of transducer (i.e., curvature and element pitch) and the transmit focal depth and was found to be 33. The optimal sound speed was estimated to be 1500 m/s, which was closed to the sound speed in water at room temperature [29]. All images were logarithmically compressed with a dynamic range of 40 dB. As shown in Figure 2, improved lateral resolution in the SA-VS image can be readily recognized under visual assessment.

**Figure 3:**
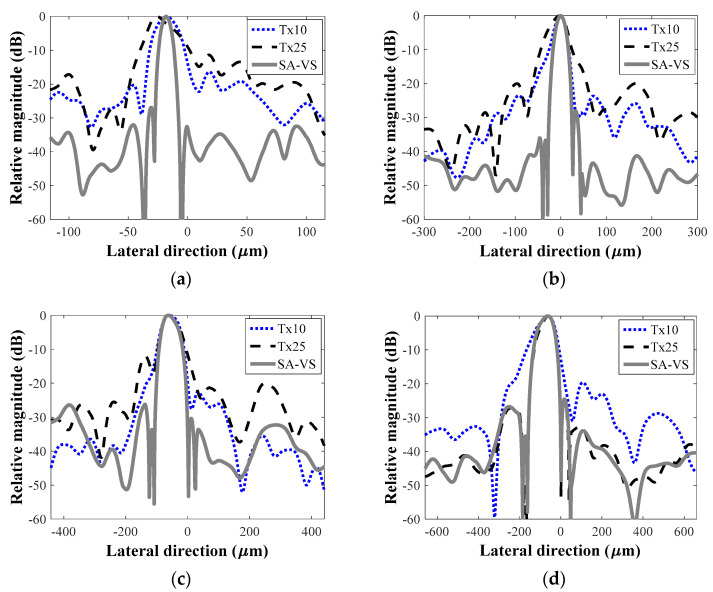
Lateral beam profiles from each method at depths of (**a**) 5 mm, (**b**) 14 mm, (**c**) 18 mm, and (**d**) 24 mm, respectively.

For quantitative comparison, the lateral beam profiles at depths of 4, 14, 18, and 24 mm were measured and plotted in Figure 3. As can be seen, the SA-VS method produced not only improved lateral resolutions but also decreased sidelobe levels compared to those from the conventional one-way dynamic focusing methods. At the depth of 24 mm, similar beam profiles were obtained from the SA-VS and the conventional method with the focal depth of 25 mm. The −6 dB lateral resolutions and first sidelobe levels are summarized in Table 1. As listed in Table 1, the −6 dB lateral resolutions and the sidelobe levels in the SA-VS method were improved at all imaging depths. The lateral resolutions were improved by more than 10% (maximally 65%) except at the depth of 24 mm where similar beam profiles were produced between the conventional (Tx25) and SA-VS methods. Considerable reductions in the sidelobe level were obtained by the SA-VS method; minimal and maximal enhancements were, respectively, 4.4 and 15.6 dB.

**Figure 4:**
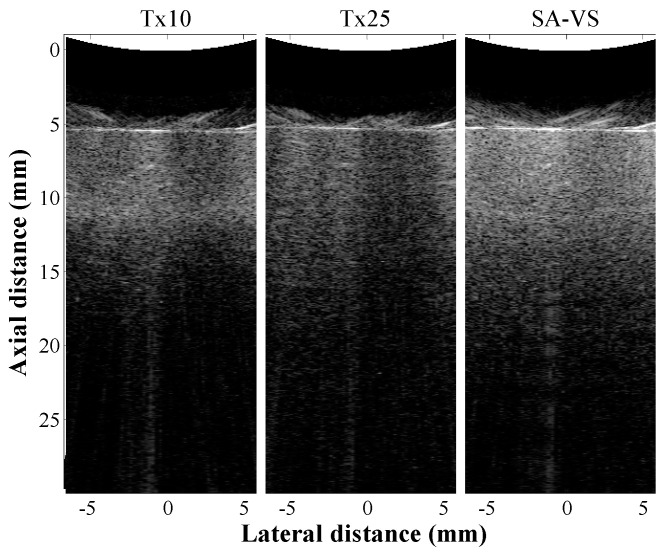
B-mode images of agar phantom obtained by the conventional method with transmit focal depths of 10 mm and 25 mm and the SA-VS method, respectively. The number of sub-aperture used in synthesis in the SA-VS was 33.

**Figure 5:**
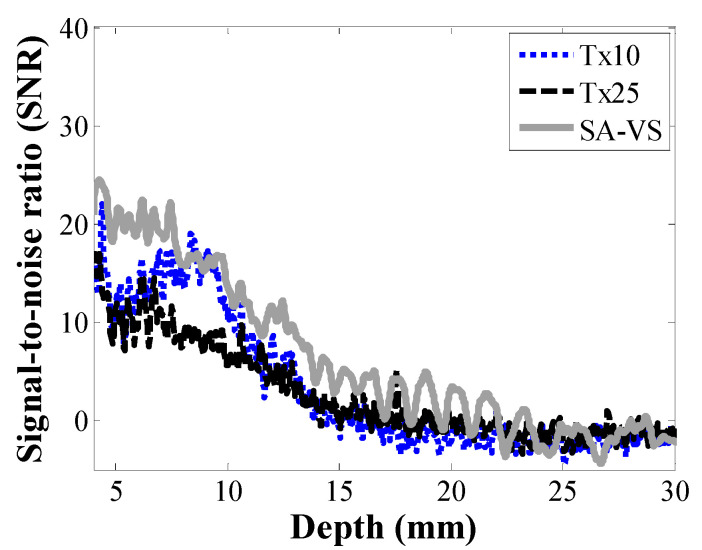
Measured SNRs for each method as a function of depth.

B-mode images of a custom-made agar phantom are shown in Figure 4. In conventional imaging, the transmit focal depths were 10 (Tx10) and 25 mm (Tx25), respectively. For the SA-VS imaging, a transmit focal depth was 10 mm, and the number of sub-aperture for synthesis was 33. As shown in Figure 4, the SA-VS produced speckle patterns with uniform brightness. The penetration depth was also increased in the SA-VS. The measured SNR curves for each method as a function of depth are shown in Figure 5. The mean power at each depth was computed from 20 scanlines at the center. Consistent with the visual assessment, the SA-VS method improved the SNR for the entire imaging depth compared to other methods. In the conventional method with the transmit focusing at 10 mm, the maximum SNR was achieved around 10 mm, and the SNR sharply decreased after the focal depth due to diffraction and attenuation. Due to the high attenuation, the SNR curve with the conventional method (Tx25) could not produce a peak at 25 mm. From the curves, the penetration depths were determined to be 16 and 23 mm for the conventional and SA-VS methods, respectively.

**Figure 6:**
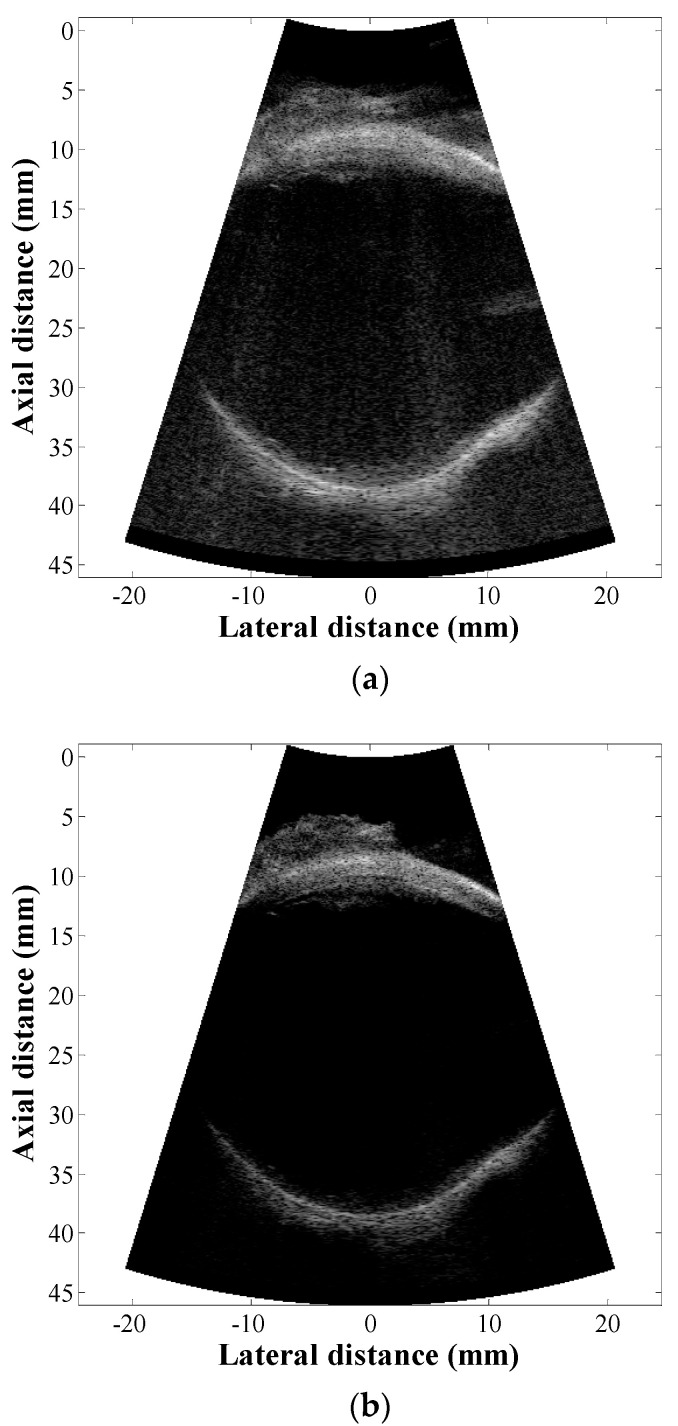
B-mode images of an excised bovine eye by (**a**) the conventional and (**b**) SA-VS methods. In the conventional method, a transmit focal depth was 35 mm while it was 10 mm for the SA-VS method. The number of sub-aperture used in synthesis in the SA-VS was 33.

Figure 6 shows B-mode images of the bovine eye with the conventional and SA-VS methods. The image was acquired by avoiding the lens exhibiting high attenuation and propagation sound speed [30]. In this experiment, the optimal sound speed was estimated to be 1520 m/s and was well agreed with the previously reported value (i.e., 1513 m/s in the vitreous body) [30]. The transmit focal depth was 35 mm in the conventional method while it was 10 mm for the SA-VS method. The number of sub-aperture used in synthesis was 33. The images were rendered without any further processing such as filtering and post-image processing. Consistent with the results of phantom experiments, as shown in Figure 6, the SA-VS produced improved image quality compared to that by the conventional method. In the conventional method, the image was considerably blurred, especially in the anterior segment. On the other hand, the anterior and posterior segments were clearly visualized due to the enhancement of resolution. In addition, increased SNR in the SA-VS imaging allowed for visualizing structures that were ambiguous by noise in the conventional imaging; scattering from the vitreous body is clearly visualized proximal to the retina in the SA-VS imaging.

In ophthalmic imaging, detailed information such as vitreous detachment, hemorrhage, and intraocular foreign body is important to diagnose and manage ocular emergencies, which can be achieved by increasing the center frequency of ultrasound imaging. Although the vitreous body is a gel-like substance and is known as acoustically transparent, ultrasound imaging with higher frequency (>20 MHz) still suffers from a high attenuation [31]. The method presented in the paper could resolve these problems (lower resolution at out-of-focus regions and SNR) and would be useful to diagnose and manage ocular emergencies.

Tissue motion and phase aberration are primary factors that limit the effectiveness of SA imaging [32]. Phase aberration correction based on correlation measurements would be the best solution. However, it requires a significant amount in computing correlation, which would be difficult to implement in real time. As a remedy, a method of estimating an optimal sound speed that can reduce the phase aberration artifacts has been proposed [28], which was used in the paper. Although it can partially resolve the problem, a previous study showed its potential in improving image quality in clinical practices [22]. Moreover, the effect of phase aberration in the ophthalmic SA imaging would be insignificant since the most of eye consists of a vitreous body that is a homogeneous medium.

## 4. Conclusions

Ultrasound imaging with the SA-VS was illustrated and showed its potential for ophthalmic imaging. The performances of the SA-VS method were evaluated through phantom and ex vivo experiments. The experimental results demonstrated that the SA-VS method can improve both lateral resolution and SNR. It was demonstrated that SA-VS imaging has the potential to deliver more additional significant information with diagnostic analytics compared to the conventional imaging method. Recent advances in electronics such as high-performance FPGA or GPU would support its high computational load and accelerate the commercialization of the SA-VS method. Further clinical evaluations of the SA-VS imaging need to be followed under various disease conditions to become an essential imaging tool for ophthalmic imaging.

## Figures and Tables

**Table 1:** The −6 dB lateral resolutions and first sidelobe levels for conventional Tx10, Tx25, and SA-VS.

	−6 dB Lateral Resolution (μm)/First Sidelobe Level (dB)			
	5 mm	14 mm	18 mm	24 mm
Conventional Tx10	24.0/−16.4	32.2/−23.7	71/−22.5	119.3/−19.6
Conventional Tx25	29.6/−11.4	51.3/−20.0	72.6/−12	106.0/−24.6
SA-VS	10.2/−27.0	26.9/−28.1	64.3/−26.2	105.5/−24.6

## Data Availability

Not applicable.
